# CircFOXM1 promotes the proliferation, migration, invasion, and glutaminolysis of glioblastoma by regulating the miR-577/E2F transcription factor 5 axis

**DOI:** 10.17305/bjbms.2021.6028

**Published:** 2021-10-12

**Authors:** Xuhui Fan, Meng Liu, Li Fei, Zhihui Huang, Yufeng Yan

**Affiliations:** Department of Neurosurgery, Jinshan Hospital Affiliated to Fudan University, Shanghai, China

**Keywords:** Glioblastoma, circFOXM1, miR-577, E2F transcription factor 5

## Abstract

Circular RNA (circRNA) is a key regulator of tumor progression. However, the role of circFOXM1 in glioblastoma (GBM) progression is unclear. The aim of this study was to investigate the role of circFOXM1 in GBM progression. The expression levels of circFOXM1, miR-577, and E2F transcription factor 5 (E2F5) were examined by real-time quantitative polymerase chain reaction. Cell counting kit 8 assay, EdU staining, and transwell assay were used to detect cell proliferation, migration, and invasion. The levels of glutamine, glutamate, and α-ketoglutarate were determined to evaluate the glutaminolysis ability of cells. Protein expression was tested by Western blot analysis. Dual-luciferase reporter assay, RNA pull-down assay, and RNA immunoprecipitation assay were employed to verify the interaction between miR-577 and circFOXM1 or E2F5. Mice xenograft model for GBM was constructed to perform *in vivo* experiments. Our results showed that circFOXM1 was highly expressed in GBM tumor tissues and cells. Silencing of circFOXM1 inhibited GBM cell proliferation, migration, invasion, glutaminolysis, as well as tumor growth. MiR-577 could be sponged by circFOXM1, and its inhibitor could reverse the suppressive effect of circFOXM1 downregulation on GBM progression. E2F5 was a target of miR-577, and the effect of its knockdown on GBM progression was consistent with that of circFOXM1 silencing. CircFOXM1 positively regulated E2F5 expression, while miR-577 negatively regulated E2F5 expression. In conclusion, our data confirmed that circFOXM1 could serve as a sponge of miR-577 to enhance the progression of GBM by targeting E2F5, which revealed that circFOXM1 might be a biomarker for GBM treatment.

## INTRODUCTION

Glioblastoma (GBM) is the most malignant type of glioma with rapid and diffuse infiltrative growth [1,2]. Despite great efforts in treatment, the prognosis of GBM patients is still poor with a typical survival of only about 15 months [3,4]. GBM often causes a variety of neurological symptoms, which greatly reduces the life quality of patients [5,6]. It is necessary to better understand the molecular pathogenesis of GBM and develop effective targeted therapies for GBM. Glutaminolysis is an important process to maintain energy metabolism and homeostasis of cancer cells, and it has been found to be closely related to the proliferation of GBM [7,8]. Therefore, elucidating the molecular mechanisms affecting GBM proliferation, metastasis, and glutaminolysis is expected to provide effective targets for GBM therapy.

Circular RNA (circRNA) is a newly confirmed special class of non-coding RNA with stable expression [9,10]. In recent years, studies have found that circRNA plays an essential regulatory role in the development of many diseases, including cancer [11,12]. This not only gives us a better understanding of circRNA but also provides us the new research directions for the diagnosis, treatment, and prevention of diseases. For example, Zhu et al. suggested that circENTPD7 silencing could suppress the motility and proliferation of GBM, which provided a new biomarker for the targeted therapy of GBM [13]. Lv et al. reported that circ-EPB41L5 played a tumor-suppressor role in GBM, which could inhibit GBM proliferation and metastasis [14]. In addition, circ-0001801 also was found to be upregulated in GBM, and its knockdown was considered to be an effective way to hinder the progression of GBM [15]. Therefore, circRNA might be an important regulator for GBM progression. In our study, we screened the differentially expressed circRNA in GBM tumor tissues and normal tissues in GEO database (accession: GSE109569) and found that hsa_circ_0025033 (circFOXM1) was notably upregulated in GBM tumor tissues. In the previous studies, cirFOXM1 was found to be highly expressed in ovarian cancer and papillary thyroid carcinoma, and had been proven to promote the malignant progression of cancers [16-18]. However, the current role of circFOXM1 in GBM is unclear.

Many studies have confirmed that circRNA can be used as “miRNA sponge” to regulate downstream gene expression to affect cell biological functions [19,20]. MiR-577 was found to be downregulated in GBM and played a negative role in GBM proliferation and metastasis [21,22]. E2F transcription factor 5 (E2F5) has been discovered to be upregulated in GBM and is involved in regulating cell proliferation and tumor growth [23,24]. Through bioinformatics analysis, we discovered that circFOXM1 could sponge miR-577 and miR-577 could target E2F5. Therefore, we proposed the hypothesis that circFOXM1 mediated GBM progression through the miR-577/E2F5 axis and verified that in this study.

## MATERIALS AND METHODS

### Patients and tissues collection

Tumor tissues and adjacent normal tissues were collected from 40 GBM patients who underwent surgery at the Jinshan Hospital Affiliated to Fudan University. All tissues were stored at −80. The clinicopathologic features of GBM patients are shown in Table 1. For this study, we obtained written informed consent from each patient. Our study was approved by the Ethics Committee of Jinshan Hospital Affiliated to Fudan University (reference number HB20180013) and carried out according to the guidelines of the Declaration of Helsinki.

**TABLE 1:**
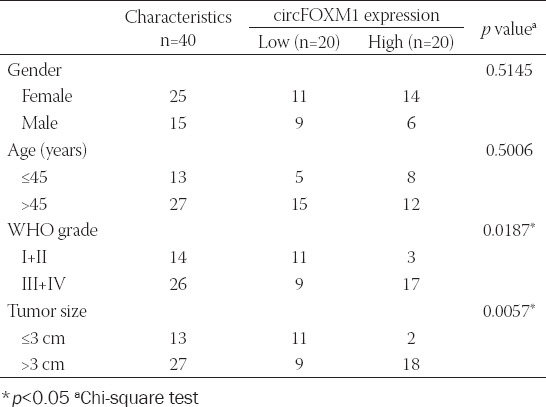
Relationship between circFOXM1 expression and clinicopathologic features of GBM patients

### Cell culture

Human GBM cell lines (U251, LN229, T98, and A172) and normal astrocytes (NHAs) were purchased from Procell (Wuhan, China). All cells were cultured in Dulbecco’s modified Eagle’s medium (DMEM) medium (Gibco, Grand Island, NJ, USA) containing 10% fetal bovine serum (FBS) (Gibco) and 1% penicillin-streptomycin solution (Procell) at 37 with 5% CO_2_ incubator.

### Real-time quantitative polymerase chain reaction (RT-qPCR)

The RNA was obtained using TRIzol reagent (Invitrogen, Carlsbad, CA, USA), and cDNA was synthesized using PrimeScript RT Master Mix (Takara, Dalian, China). Based on the manufacturer’s instructions of SYBR Green (Invitrogen), RT-qPCR was carried out on a PCR system. Relative expression was calculated by the 2^−ΔΔCt^ method and normalized by β-actin or U6. The primers were listed as below: circFOXM1, F 5’-GGTGTGAGCCAGCTTGAGA-3’, R 5’-GACGGGGGCTAGTTTTCATT-3’; FOXM1, F 5’-CGTCGGCCACTGATTCTCAAA-3’, R 5’-GGCAGGGGATCTCTTAGGTTC-3’; miR-577, F 5’-TGCGGTAGATAAAATATTGG-3’, R 5’-CCAGTGCAGGGTCCGAGGT-3’; E2F5, F 5’-GCCCGTGGTTTTTCCTGTTC-3’, R 5’-CCACTAATAGATCCTGCTGAAGA-3’; β-actin, F 5’-ATAGCACAGCCTGGATAGCAACGTAC-3’, R 5’-CACCTTCTACAATGAGCTGCGTGTG-3’; and U6, F 5’-CGCTTCGGCAGCACATATACTA-3’, R 5’-CGCTTCACGAATTTGCGTGTCA-3’.

### RNase R assay

After extracting RNA from LN229 and A172 cells, the RNA was incubated with RNase R (Lucigen, Middleton, WI, USA) for 10 min. Using RNA not incubated with RNase R as the negative control (Mock), the expression of circFOXM1 and linear FOXM1 in two RNAs was determined by RT-qPCR.

### Actinomycin D (ActD) assay

LN229 and A172 cells were incubated with ActD (AAT Bioquest, Sunnyvale, CA, USA) for certain times (0, 4, 8, 12, and 24 hours). Then, the RNA was isolated using TRIzol reagent, and the expression of circFOXM1 and linear FOXM1 was examined by RT-qPCR.

### Cell transfection

LN229 and A172 cells were transfected with circFOXM1 and E2F5 small interference RNA (siRNA) (si-circFOXM1 and si-E2F5), lentiviral short hairpin RNA against circFOXM1 (sh-circFOXM1), miR-577 mimic or inhibitor (miR-577 or anti-miR-577), the pcDNA overexpression vectors of circFOXM1 and E2F5 (pcDNA-circFOXM1 and pcDNA-E2F5), or their negative controls using Lipofectamine 3000 (Invitrogen). The concentrations of oligonucleotides were 50 nM, and the concentrations of vectors were 4 μg.

### Cell proliferation assay

For cell counting kit 8 (CCK8) assay, LN229 and A172 cells were reseeded into 96-well plates. At the indicated times, each well was added with 10 μL CCK8 reagent (Beyotime, Shanghai, China). After incubated for 4 hours, the optical density value was determined by a microplate reader at 450 nm. For EdU staining, LN229 and A172 cells were stained by EdU Cell proliferation Detection Kit (Beyotime) according to the kit instructions.

### Cell migration and invasion assays

Transwell chambers (Corning Inc., Corning, NY, USA) were used for detecting cell migration and invasion, in which the chambers used for cell invasion were pre-coated with a Matrigel. LN229 and A172 cells were seeded into the upper chambers with DMEM medium. The lower chambers were filled with DMEM medium containing 10% FBS. After 24 h, the transferred cells were fixed with paraformaldehyde and stained with crystal violet (all from Sigma-Aldrich, St. Louis, MO, USA). The field of view was randomly selected under the microscope (100×) to count the number of cells.

### Measurement of cell glutaminolysis

Glutamine and Glutamate Determination Kit and α-ketoglutarate (α-KG) Assay Kit were bought from Sigma-Aldrich. According to the manufacturer’s instruction, the levels of glutamine, glutamate, and α-KG were determined.

### Western blot (WB) analysis

Briefly, cells and tissues were lysed by RIPA lysis buffer (Beyotime) to extract total protein, and the protein was quantified by BCA kit (Beyotime). Equal amounts of protein were separated by 10% SDS-PAGE and transferred to PVDF membranes (Invitrogen). After blocking with 5% skim milk, the membranes were probed with primary antibodies against E2F5 (1:10,000, Invitrogen), CyclinD1 (1:1000, Invitrogen), ASCT2 (1:600, Invitrogen), GLS1 (1:1000, Invitrogen), and β-actin (1:10,000, Invitrogen) at 4°C overnight. Then, the membranes were incubated with respective secondary antibodies (1:20,000, Invitrogen) for 1 hour at room temperature. The immunoblots were visualized by BeyoECL Plus ECL Reagent (Beyotime).

### Dual-luciferase reporter assay

The sequences of circFOXM1 or E2F5 3’UTR containing the binding sites (WT) for miR-577 and its corresponding mutant sites (MUT) were cloned and inserted into the pmirGLO reporter vector. LN229 and A172 cells were co-transfected with the reporter vectors and miRNA mimic or miR-NC. After 48 hours, the relative luciferase activity was evaluated using Dual-Luciferase Reporter Gene Assay Kit (Beyotime).

### RNA pull-down assay

The biotin-labeled miR-577 probe and mutated probe (Bio-miR-577 and Bio-miR-577-MUT) or negative control probe (Bio-miR-NC) were obtained from GenePharma (Shanghai, China). LN229 and A172 cells were transfected with the probes for 48 hours. Then, the cells were lysed, and the cell lysates were collected and then incubated with Dynabeads M-280 Streptavidin (Invitrogen). After extracted RNA from the probe magnetic bead complex, the enrichments of circFOXM1 and E2F5 were measured by RT-qPCR.

### RNA immunoprecipitation (RIP) assay

After transfection for 48 hours, LN229 and A172 cells were lysed, and the cells lysates were obtained. Subsequently, the cell lysates were incubated overnight with magnetic beads (Pierce, Rockford, IL, USA) containing anti-Ago2 or anti-IgG. After purified the immunoprecipitated RNA, the RNA levels of circFOXM1, miR-577, and E2F5 were assessed by RT-qPCR.

### Animal experiments

A172 cells were transfected with sh-NC and sh-circFOXM1 for 48 hours. After that, the transfected cells (2 × 10^6^) were suspended with PBS and then injected subcutaneously into the left flank of BALB/c male nude mice (Sebiona, Guangzhou, China). The length and width of the tumors were measured with a Vernier caliper every 3 days, and the changes of tumor volume were recorded. After 24 days, the mice were euthanized and their tumors were weighed. The expression levels of circFOXM1, miR-577, and E2F5 in the tumors were detected by RT-qPCR and WB analysis, respectively. Paraffin sections were prepared from tumor tissue, and then, Ki67, E2F5, and MMP9 immunohistochemical (IHC) staining was performed on the sections using the SP Kit (Invitrogen) with corresponding antibodies (Invitrogen). All experiments were approved by the Animal Ethics Committee of Jinshan Hospital Affiliated to Fudan University (reference number 2019AN0003) and were performed according to the Guide for the Care and Use of Laboratory Animals.

### Statistical analysis

All statistical analyses were performed using GraphPad Prism 8.0. The results were presented as mean ± SD. Differences between groups were compared using Student’s *t*-test or one-way analysis of variance followed by Tukey *post hoc* test. The correlation analysis was conducted using the Pearson correlation analysis. *p* < 0.05 was considered to be statistically significant.

## RESULTS

### The expression and validation of circFOXM1 in GBM tumor tissues and cells

**FIGURE 1:**
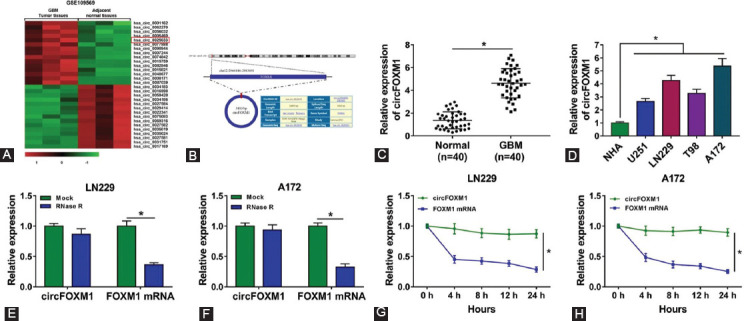
The expression and validation of circFOXM1 in glioblastoma (GBM) tissues and cells. (A) Heat map showed the differentially expressed circular RNA in GBM tumor tissues and adjacent normal tissues; (B) The formation of circFOXM1 was presented; (C) The expression of circFOXM1 in GBM tumor tissues (GBM) and adjacent normal tissues (normal) was determined by real-time quantitative polymerase chain reaction (RT-qPCR); (D) RT-qPCR was employed to measure circFOXM1 expression in normal astrocytes cells and GBM cell lines (U251, LN229, T98, and A172); RNase R assay (E-F) and ActD assay (G-H) were used to evaluate the stability of circFOXM1 and FOXM1. *p < 0.05.

Based on the cutoff criteria of values (|log2 fold change| >1 and *p* < 0.05), we found a total of 15 upregulated circRNAs and 15 downregulated circRNAs in GBM tumor tissues, among which circFOXM1 was significantly upregulated (Figure 1A). The circBase information revealed that circFOXM1 is located at chr12:2966846-2983691 and is derived from the FOXM1 genome (Figure 1B). By detecting the expression of circFOXM1, we found that circFOXM1 was markedly highly expressed in GBM tumor tissues compared to adjacent normal tissues (Figure 1C), and it also was obviously overexpressed in four GBM cell lines (U251, LN229, T98, and A172) compared with that in NHA cells, especially in LN229 and A172 cells (Figure 1D). By analyzing the relationship between circFOXM1 expression and clinicopathologic features in GBM patients, we found that the high expression of circFOXM1 was related to WHO grade and tumor size in patients (Table 1). Using the RNase R assay and ActD assay, we found that circFOXM1 could resist the digestion of RNase R and was more stable than linear FOXM1 mRNA (Figure 1E-H), confirming that circFOXM1 was indeed a circRNA.

### Silencing of circFOXM1 inhibited the proliferation, migration, invasion, and glutaminolysis of GBM cells in vitro

**FIGURE 2:**
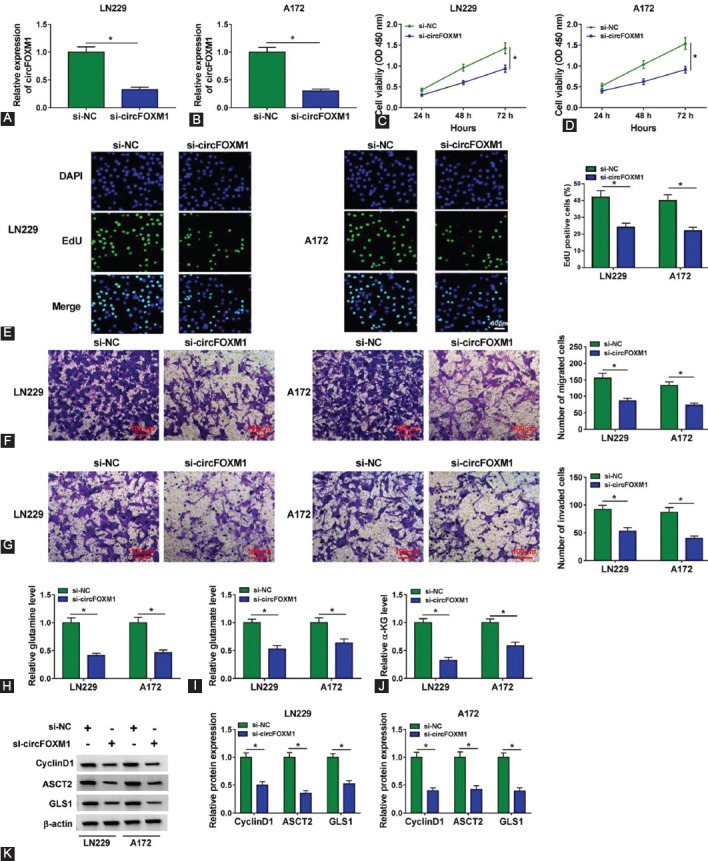
Silencing of circFOXM1 inhibited the progression of glioblastoma cells *in vitro*. LN229 and A172 cells were transfected with si-NC or si-circFOXM1. (A-B) The expression of circFOXM1 was detected by real-time quantitative polymerase chain reaction; CCK8 assay (C-D) and EdU staining (E) were used to measure the proliferation of cells; (F-G) The migration and invasion of cells were determined by transwell assay; (H-J) The levels of glutamine, glutamate, and α-KG were examined by Glutamine and Glutamate Determination Kit and α-KG Assay Kit, respectively; (K) WB analysis was performed to assess the protein expression of CyclinD1, ASCT2, and GLS1. *p < 0.05.

To illuminate the role of circFOXM1 in GBM progression, we performed the loss-of-function experiments using si-circFOXM1. After transfected with si-circFOXM1 into LN229 and A172 cells, we confirmed that the expression of circFOXM1 was notably decreased (Figure 2A and B). CCK8 assay and EdU staining results indicated that cell viability and EdU-positive cells were significantly suppressed by circFOXM1 knockdown (Figure 2C and E), confirming that circFOXM1 might promote GBM proliferation. Furthermore, the numbers of migrated and invaded LN229 and A172 cells also were inhibited after silencing of circFOXM1, suggesting that circFOXM1 had an active role in the migration and invasion of GBM cells (Figure 2F and G). In addition, we also analyzed the glutaminolysis ability of GBM cells and found that circFOXM1 knockdown resulted in decreased levels of glutamine, glutamate, and α-KG (Figure 2H and J). Furthermore, silenced circFOXM1 also repressed the protein expression of proliferation marker CyclinD1, glutamine transporter ASCT2, and glutamine rate-limiting enzyme GLS1 in LN229 and A172 cells (Figure 2K).

### CircFOXM1 served as a molecular sponge of miR-577

**FIGURE 3:**
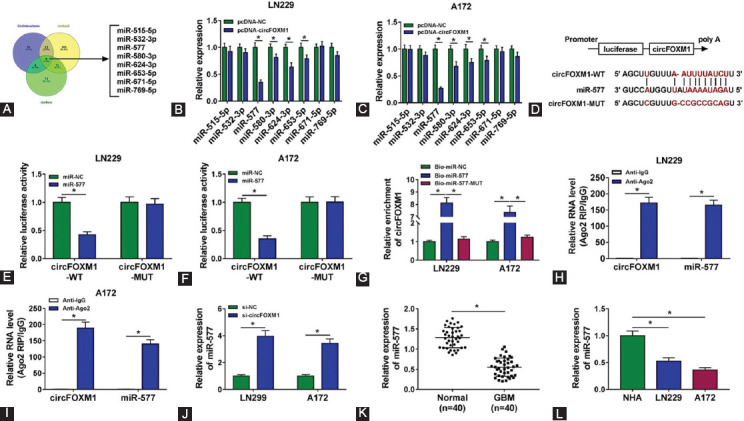
CircFOXM1 sponged miR-577. (A) The predicted miRNAs that could be targeted by circFOXM1 were shown; (B-C) After transfected with pcDNA-circFOXM1 or pcDNA-NC into LN229 and A172 cells, the expression levels of candidate miRNAs were determined by real-time quantitative polymerase chain reaction (RT-qPCR); (D) The putative binding sites of miR-577 in circFOXM1 were presented; Dual-luciferase reporter assay (E-F), RNA pull-down assay (G), and RIP assay (H-I) were used to verify the interaction between circFOXM1 and miR-577; (J) The expression of miR-577 was detected by RT-qPCR in LN229 and A172 cells transfected with si-NC or si-circFOXM1. RT-qPCR was performed to test the expression of miR-577 in glioblastoma (GBM) tumor tissues (GBM) and adjacent normal tissues (normal) (K), as well as in normal astrocytes cells and GBM cell lines (LN229 and A127) (L). *p < 0.05.

To explore the potential molecular mechanisms of circFOXM1, we used the starBase, circbank, and CircInteractome software to predict the miRNAs that could be complementary to circFOXM1. The results showed that a total of eight miRNAs could be targeted by circFOXM1 (Figure 3A). After preliminary screening, we found that circFOXM1 overexpression had a strong inhibitory effect on miR-577 expression in both LN229 and A172 cells (Figure 3B and C). Therefore, miR-577 was selected for functional verification. According to the binding sites of circFOXM1 and miR-577, we constructed the circFOXM1-WT/MUT reporter vectors (Figure 3D). Dual-luciferase reporter assay results indicated that miR-577 overexpression could obviously suppress the luciferase activity of circFOXM1-WT reporter vector, while not affect that of circFOXM1-MUT reporter vector (Figure 3E and F). Besides, RNA pull-down assay results showed that circFOXM1 could be enriched in the Bio-miR-577 probe compared with that in the Bio-miR-NC and Bio-miR-577-MUT probes (Figure 3G). Moreover, RIP assay also suggested that the enrichments of circFOXM1 and miR-577 were markedly enhanced in anti-Ago2 (Figure 3H and I). These results verified that circFOXM1 could interact with miR-577 in GBM cells. Furthermore, we also discovered that circFOXM1 knockdown could promote the expression of miR-577 in LN229 and A172 cells (Figure 3J). In addition, miR-577 was found to be downregulated in GBM tumor tissues and cell lines (Figure 3K and L).

### MiR-577 inhibitor reversed the inhibitory effect of circFOXM1 knockdown on GBM progression

**FIGURE 4:**
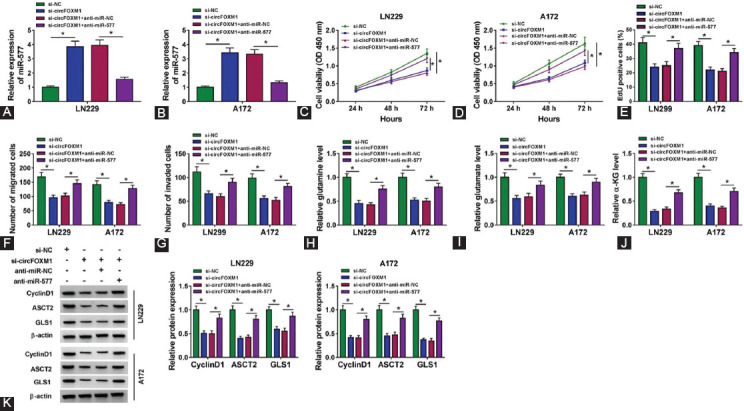
Effects of circFOXM1 knockdown and miR-577 inhibitor on glioblastoma progression. LN229 and A172 cells were transfected with si-NC, si-circFOXM1, si-circFOXM1 + anti-miR-NC, or si-circFOXM1 + anti-miR-577. (A-B) Real-time quantitative polymerase chain reaction was used to test the expression of miR-577; The proliferation of cells was evaluated by CCK8 assay (C-D) and EdU staining (E); (F-G) Transwell assay was used to measure the migration and invasion of cells; (H-J) Glutamine and Glutamate Determination Kit and α-KG Assay Kit were employed to determine the levels of glutamine, glutamate, and α-KG, respectively; (K) The protein expression levels of CyclinD1, ASCT2, and GLS1 were detected by WB analysis. *p < 0.05.

The rescue experiment was carried out to confirm whether circFOXM1 regulated GBM progression through targeting miR-577. After cotransfected si-circFOXM1 and anti-miR-577 into LN229 and A172 cells, the promotion effect of circFOXM1 silencing on miR-577 expression was reversed by miR-577 inhibitor, showing that the transfection was effective (Figure 4A and B). By measuring cell viability, EdU-positive cells, and the numbers of migrated and invaded cells, we found that the suppressive effect of circFOXM1 silencing on the proliferation, migration, and invasion of GBM cells could be reversed by miR-577 inhibitor (Figure 4C-G). In addition, the levels of glutamine, glutamate, and α-KG restrained by circFOXM1 knockdown also were inverted by the inhibition of miR-577 (Figure 4H and J). Moreover, miR-577 inhibitor also reversed the decreasing function of circFOXM1 downregulation on the protein levels of CyclinD1, ASCT2, and GLS1 in LN229 and A172 cells (Figure 4K). Therefore, our data suggested that circFOXM1 regulates GBM progression by sponging miR-577.

### E2F5 was targeted by miR-577

**FIGURE 5:**
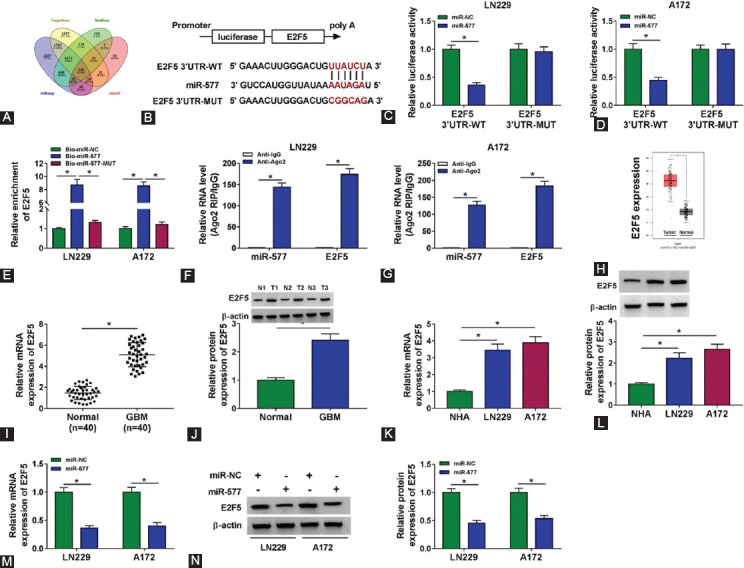
MiR-577 targeted E2F5. (A) The candidate genes of miR-577 were predicted by the miRmap, TargetScan, starBase, and microT software; (B) The putative binding sites of miR-577 in E2F5 3’UTR were shown; The interaction between miR-577 and E2F5 was confirmed by dual-luciferase reporter assay (C-D), RNA pull-down assay (E), and RIP assay (F-G); (H) TCGA COAD database showed the expression of E2F5 in glioblastoma (GBM) tumor tissues and adjacent normal tissues; (I-J) The mRNA and protein expression levels of E2F5 were determined by real-time quantitative polymerase chain reaction (RT-qPCR) and WB analysis; (K-L) RT-qPCR and WB analysis were used to measure the mRNA and protein expression levels of E2F5 in normal astrocytes cells and GBM cell lines (LN229 and A127); (M-N) After transfected with miR-NC and miR-577 mimic into LN229 and A172 cells, the mRNA and protein expression levels of E2F5 were assessed by RT-qPCR and WB analysis. *p < 0.05.

In addition, miRmap, TargetScan, starBase, and microT software were used to predict the targeted genes of miR-577, and the results showed that a total of 135 genes could interact with miR-577 (Figure 5A). Among them, we found a gene, E2F5, which had been shown to play a pro-oncogenic role in various cancers, so E2F5 was selected for our study. The E2F5 3’UTR-WT/MUT reporter vectors were constructed to perform dual-luciferase reporter assay (Figure 5B). The results showed that the luciferase activity of E2F5 3’UTR-WT reporter vector could be reduced by miR-577 overexpression (Figure 5C and D). Meanwhile, we also found a significant enrichment of E2F5 in the Bio-miR-577 probe (Figure 5E). The results of RIP assay suggested that the levels of miR-577 and E2F5 were remarkably increased in anti-Ago2 (Figure 5F and G). In the TCGA COAD database, we discovered that E2F5 expression was remarkably overexpressed in GBM tissues (Figure 5H). In our study, we detected the expression of E2F5 and found that compared with adjacent normal tissues and NHA cells, the mRNA and protein expression levels of E2F5 were obviously upregulated in GBM tumor tissues and cell lines (Figure 5I-L). In addition, at the mRNA level and protein level, we also found that the expression of E2F5 could be repressed by miR-577 overexpression (Figure 5M and N).

### E2F5 expression was negatively regulated by miR-577 and positively regulated by circFOXM1

**FIGURE 6:**
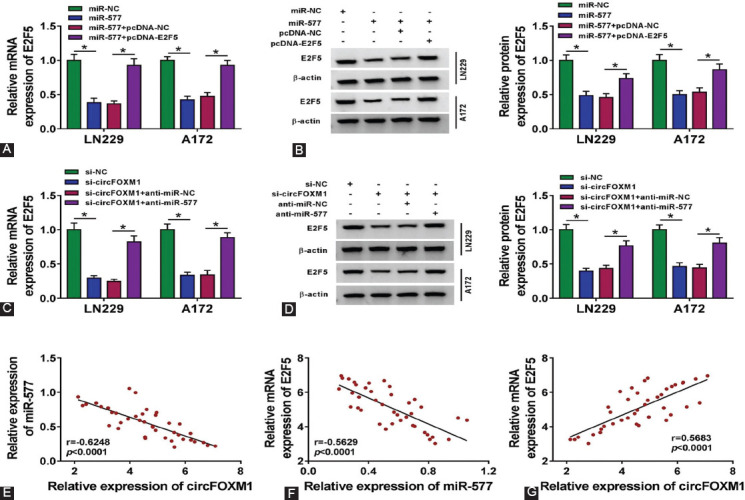
MiR-577 and circFOXM1 regulate E2F5 expression. (A-B) LN229 and A172 cells were transfected with miR-NC, miR-577, miR-577 + pcDNA-NC, or miR-577 + pcDNA-E2F5. The mRNA and protein expression levels of E2F5 were determined by real-time quantitative polymerase chain reaction (RT-qPCR) and WB analysis; (C-D) LN229 and A172 cells were transfected with si-NC, si-circFOXM1, si-circFOXM1 + anti-miR-NC, or si-circFOXM1 + anti-miR-577. The mRNA and protein expression levels of E2F5 were determined by RT-qPCR and WB analysis; (E-G) Pearson correlation analysis was used to evaluate the correlations among circFOXM1, miR-577, and E2F5 in glioblastoma tumor tissues. *p < 0.05.

To confirm the regulation of miR-577 on E2F5 expression, we cotransfected miR-577 mimic and pcDNA-E2F5 into LN229 and A172 cells. Through measuring the mRNA and protein expression levels of E2F5, we discovered that E2F5 overexpression could reverse the inhibitory effect of miR-577 overexpression on E2F5 expression (Figure 6A and B). In addition, circFOXM1 silencing could suppress the mRNA and protein expression of E2F5, while this effect could be reversed by miR-577 inhibitor (Figure 6C and D). Correlation analysis results indicated that miR-577 expression was negatively correlated with circFOXM1 and E2F5 expression (Figure 6E and F), and circFOXM1 expression was positively correlated with E2F5 expression in GBM tissues (Figure 6G). These results revealed that circFOXM1 positively regulated E2F5 expression by sponging miR-577.

### E2F5 knockdown hindered the proliferation, migration, invasion, and glutaminolysis of GBM cells *in vitro*

**FIGURE 7:**
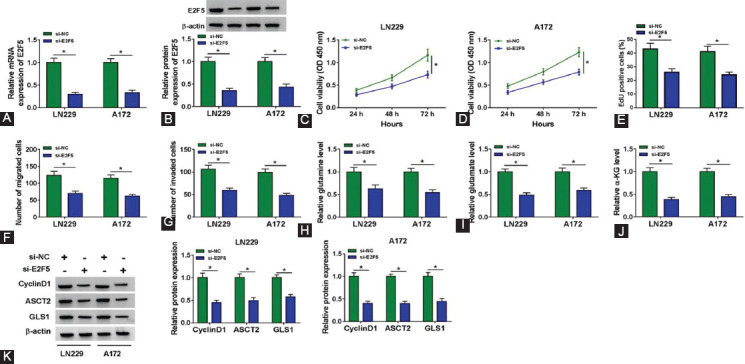
E2F5 knockdown hindered the progression of glioblastoma cells *in vitro*. LN229 and A172 cells were transfected with si-NC or si-E2F5. (A-B) The mRNA and protein expression levels of E2F5 were measured by real-time quantitative polymerase chain reaction and WB analysis; The proliferation of cells was determined using CCK8 assay (C-D) and EdU staining (E); (F-G) The migration and invasion of cells were assessed by transwell assay; (H-J) The levels of glutamine, glutamate, and α-KG were examined using Glutamine and Glutamate Determination Kit and α-KG Assay Kit, respectively; (K) The protein expression levels of CyclinD1, ASCT2, and GLS1 were tested by WB analysis. *p < 0.05.

To determine that circFOXM1 regulates GBM progression by mediating E2F5 expression, E2F5 expression was silenced using si-E2F5. The reduced E2F5 expression confirmed that our transfection was successful (Figure 7A and B). The results of CCK8, EdU staining, and transwell assays suggested that after E2F5 silencing, cell viability, EdU-positive cells, and the numbers of migrated and invaded cells were remarkably suppressed (Figure 7C-G). Meanwhile, the levels of glutamine, glutamate, and α-KG were inhibited in the presence of si-E2F5 (Figure 7H-J). Furthermore, E2F5 silencing also restrained the protein expression of CyclinD1, ASCT2, and GLS1 in LN229 and A172 cells (Figure 7K). All data showed that E2F5 knockdown could repress the progression of GBM, which was consistent with the function of circFOXM1 downregulation on GBM progression.

### Interference of circFOXM1 suppressed GBM tumor growth *in vivo*

**FIGURE 8:**
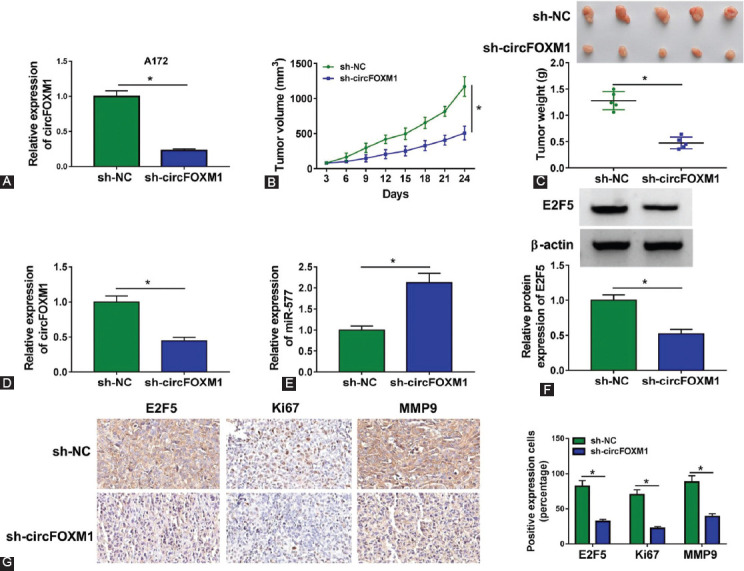
CircFOXM1 knockdown suppressed glioblastoma tumor growth *in vivo*. (A) The circFOXM1 expression was assessed by qRT-PCR in A172 cells transfected with sh-NC or sh-circFOXM1; (B-G) A172 cells transfected with sh-NC or sh-circFOXM1 were injected into nude mice. Tumor volume (B) and tumor weight (C) in each group were measured; (D-E) The expression levels of circFOXM1 and miR-577 were detected by real-time quantitative polymerase chain reaction; (F) E2F5 protein expression was measured by WB analysis; (G) IHC staining was used to assess the E2F5, Ki67, and MMP9 positive cells in the tumor tissues of each group. *p < 0.05.

**FIGURE 9:**
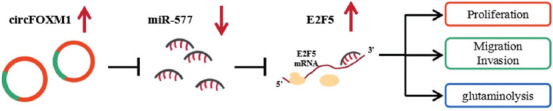
The mechanism diagram of this study. In glioblastoma (GBM), circFOXM1 promoted E2F5 expression to enhance GBM proliferation, migration, invasion, and glutaminolysis by sponging miR-577.

After transfected with sh-circFOXM1 into A172 cells, we confirmed that circFOXM1 was markedly reduced (Figure 8A). To further determine the tumorigenicity of circFOXM1, we constructed a mice xenograft model for GBM. After 24 days, we found that the tumor volume in sh-circFOXM1 group was significantly lower than that in the control group (Figure 8B), and the tumor weight in the sh-circFOXM1 group was markedly reduced compared to the control group (Figure 8C). By detecting the expression of circFOXM1, we verified that circFOXM1 expression was indeed inhibited in the sh-circFOXM1 group (Figure 8D). Anything else, miR-577 expression was notably enhanced and E2F5 expression was obviously decreased in the sh-circFOXM1 group (Figure 8E and F). In addition, IHC staining results revealed that the E2F5, Ki67, and MMP9 positive cells were markedly reduced in the tumor tissues of the sh-circFOXM1 group (Figure 8G). Hence, our data suggest that circFOXM1 promotes GBM progression by regulating the miR-577/E2F5 expression (Figure 9).

## DISCUSSION

At present, the average survival time of GBM patients after surgery, radiotherapy, and chemotherapy is only about 15 months [25,26]. In recent years, methods such as targeted therapy and immunotherapy have shown good prospects in prolonging the survival of GBM patients [27,28]. Extent of tumor resection based on fluid-attenuated inversion recovery and 5-ALA fluorescence has been validated as a feasible method, and has been clinically proven to be a stronger predictor of survival in patients with GBM [29]. More and more studies have shown that circRNA plays an important role in various pathological processes, especially in the field of cancer [11,12]. The previous studies have shown that circSMARCA5 is a tumor suppressor in GBM, which can act as a decoy for serine and arginine-rich splicing factor 1, a diagnostic immune marker for adult glioma [30,31]. Unlike this study, we suggested that circFOXM1 might play an oncogene role in GBM. Recently, circFOXM1 has been reported to participate in the progression of ovarian cancer and papillary thyroid carcinoma as a tumor promoter [16-18]. In GBM, we found that circFOXM1 had increased expression and could promote cell proliferation, migration, invasion, and glutaminolysis. In addition, animal experiments also suggested that circFOXM1 could facilitate GBM tumor growth *in vivo*. Our data revealed that circFOXM1 might play a pro-oncogenic role in GBM, which was consistent with its role in other cancers [16-18].

To elucidate the mechanism of circFOXM1 in GBM, we performed the bioinformatics prediction. We found that circFOXM1 could serve as a sponge of miR-577. The previous studies have indicated that miR-577 can act as a tumor suppressor to restrain the malignant progression of cancer, such as hepatocellular carcinoma [32], prostate cancer [33], and colorectal cancer [34]. Overexpression of miR-577 was thought to increase the permeability of the blood tumor barrier and thus enhancing the delivery of antitumor drugs to brain tumors, including glioma [35]. In this, we discovered that miR-577 expression was markedly decreased in GBM tissues and cells, which was consistent with the previous study [21,22]. The rescue experiment further illuminated that miR-577 participated in the regulation of circFOXM1 on GBM progression, which was a new discovery network.

E2F5, a member of the E2F family, has been reported to be abnormally expressed in a variety of cancers [36,37]. For example, E2F5 was upregulated in prostate cancer and could accelerate cancer migration and invasion [38]. E2F5 had been shown to promote the proliferation of neuroblastoma cells, thus proving to be an oncogene in neuroblastoma [39]. In our research, we found that E2F5 was targeted by miR-577, and its expression was positively regulated by circFOXM1 *in vivo* and *in vitro*. The positive regulation of E2F5 on GBM progression was confirmed in our study, which was consistent with the previous reported studies [23,24]. These indicated that circFOXM1 regulated GBM progression mainly by mediating E2F5 expression through targeting miR-577.

## CONCLUSION

Collectively, our results proposed that circFOXM1 was an upregulated circRNA in GBM, which could facilitate the proliferation, metastasis, and glutaminolysis of GBM through regulating the miR-577/E2F5 axis. Our research is the first to explore the role and underlying mechanism of circFOXM1 in GBM, and provides a potential molecular target for the treatment of GBM.
